# Immunization With a Live-Attenuated RH:Δ*NPT1* Strain of *Toxoplasma gondii* Induces Strong Protective Immunity Against Toxoplasmosis in Mice

**DOI:** 10.3389/fmicb.2019.01875

**Published:** 2019-08-13

**Authors:** Wen-Bin Yang, Jin-Lei Wang, Qian Gui, Yang Zou, Kai Chen, Qing Liu, Qin-Li Liang, Xing-Quan Zhu, Dong-Hui Zhou

**Affiliations:** ^1^Key Laboratory of Fujian-Taiwan Animal Pathogen Biology, College of Animal Sciences, Fujian Agriculture and Forestry University, Fuzhou, China; ^2^State Key Laboratory of Veterinary Etiological Biology, Key Laboratory of Veterinary Parasitology of Gansu Province, Lanzhou Veterinary Research Institute, Chinese Academy of Agricultural Sciences, Lanzhou, China; ^3^Department of Microbiology and Microbial Engineering, School of Life Sciences, Fudan University, Shanghai, China; ^4^College of Animal Science and Veterinary Medicine, Shanxi Agricultural University, Taigu, China; ^5^Jiangsu Co-innovation Center for the Prevention and Control of Important Animal Infectious Diseases and Zoonoses, College of Veterinary Medicine, Yangzhou University, Yangzhou, China

**Keywords:** *Toxoplasma gondii*, RH:Δ*NPT1*, immunization, live-attenuated vaccine, mice

## Abstract

Toxoplasmosis, one of the most important health-threatening diseases worldwide, is caused by *Toxoplasma gondii*, which infects a wide range of warm-blooded animals and humans, leading to enormous health and socioeconomic concerns. *T. gondii* can establish chronic infection to evade the immune response in hosts. Once a chronic infection has been established, the available treatments cannot efficiently control this stage of *T. gondii* efficiently. Moreover, the available treatments rely only on a few drugs, such as sulfapyridine and pyrimethamine, that tend to have severe side effects. Given these factors, vaccination has been considered to be the most efficient method to prevent and control this disease. However, there is currently lack of effective vaccine available for use to prevent toxoplasmosis apart form Toxovax^®^, the only available vaccine, which is used in sheep to prevent abortion. To address this problem, we knocked out the *NPT1* gene of the type I *T. gondii* strain using the CRISPR-Cas9 system, constructed a live-attenuated vaccine and evaluated its protective efficacy in a mouse model. Immunization of mice with RH:Δ*NPT1* induced a high level of *Toxoplasma*-specific IgG1, IgG2a and total IgG 42 days after immunization. There was a significant increase in the levels of cytokines in the splenocyte suspensions of RH:Δ*NPT1*-infected mice, and a mixed Th1/Th2 response was induced in the mice. Remarkably, after heterologous challenges with tachyzoites of the RH, PYS and Pru strains and cysts of the Pru strain by different infection routes, the immunized animals were protected from toxoplasmosis with a 100% survival rate, in both acute and chronic infection. In addition, compared with control mice, the Pru cyst load was clearly reduced in the brains of RH:Δ*NPT1*-infected immunization-mice. Our study demonstrated that the RH:Δ*NPT1* strain was able to evoke strong anti-*Toxoplasma* immune responses and provide effective protection against parasite strains with different levels of virulence, suggesting that the RH:Δ*NPT1* strain may represent a promising live-attenuated vaccine against toxoplasmosis, which is worthy of further evaluation in food-producing animals and in definitive feline host.

## Introduction

*Toxoplasma gondii* is an obligatory intracellular opportunistic parasitic protozoan that can infect nearly all warm-blooded animals and humans and has a worldwide distribution (Montoya and Liesenfeld, 2004; Elmore et al., 2010). *T. gondii* causes subclinical or asymptomatic infections in immunocompetent humans and animals and is also responsible for causing serious cases of encephalitis, ophthalmopathy, abortion, and stillbirth, especially in individuals with immunodeficiency or immunosuppression. Thus, this disease poses a serious threat to public health and causes great economic losses of animal husbandry (Petersen et al., 2001; Elbez-Rubinstein et al., 2009).

It has been reported that *T. gondii* can evade the immune response and establish chronic infections in most hosts, and the presently available treatments, such as sulfapyridine and pyrimethamine, do not work very well for chronic infections, leaving the survivors at a high risk of developing several neuropsychiatric disorders (Alday and Doggett, 2017; Lang et al., 2018). Although drug treatment is effective on the tachyzoite stage, it can also induce severe side effects and promote the development of drug-resistant strains (Doliwa et al., 2013). Given these factors, vaccination has been considered to be an optimal approach for prevention and control of toxoplasmosis. Therefore, developing an effective vaccine would have a large impact on public health and livestock husbandry (Zhang et al., 2015).

The progress of vaccine development has always been limited in some respects, including the complicated life cycle of the pathogen, knowledge of effective target genes and availability of appropriate technology for vaccine development, and thus there is currently lack of effective vaccine available to prevent this disease (Wang et al., 2019).

Although considerable research about the development of an anti-*T. gondii* vaccine has been carried out using various approaches with some degree of success, such as protein vaccines, DNA vaccines and inactivated vaccines, unfortunately none of these vaccines so far has provided full protection for experimental animals (Gurunathan et al., 2000; Kur et al., 2009; Zhang et al., 2013, 2015). In light of the existing anti-*T. gondii* vaccines, live attenuated vaccines have more advantages in terms of protective efficacy because they enable the vaccinated mice to develop stronger cellular and humoral responses against toxoplasmosis. Moreover, the only currently available commercial vaccine of anti-*T. gondii*, Toxovax^®^, is based on a live-attenuated *T. gondii* S48 strain, and it shows sufficient protection among sheep and goats (Buxton and Innes, 1995). This vaccine appears to demonstrate the requirements for a successful anti-*T. gondii* vaccine, namely, that it combines long-term effective protection with biosafety (the potential possibility of residual virus and transmission). However, Toxovax^®^ has some problems that need to be improved, such as its toxicity, stability and exact mechanism of action.

In several studies, researchers have attempted to use different gene-editing approaches to construct mutant strains in order to improve the protective efficiency of live-attenuated vaccine strains of *T. gondii*. Among these studies, the RH-Mic1-3KO, RH:Δ*GRA17* and ME49:Δ*ldh* strains displayed better performance in inducing protection against chronic and acute toxoplasmosis infection in a mouse model (Mévélec et al., 2010; Wang et al., 2017; Xia et al., 2018).

Recently, TgNPT1 was demonstrated to be a member of the Novel Putative Transporters (NPTs), which play an important role in the uptake of cationic amino acids by *T. gondii*. In Δ*NPT1* parasites, if the conditions of the parasite’s growth has a high ratio of [Arg]:[Lys], the TgNPT1-independent of cationic amino acids transport pathway(s) would uptake sufficient arginine to maintain growth. However, when this balance of [Arg]:[Lys] is destroyed, for example when the growth conditions in mammalian cells, the mutant parasite growth is restricted by the level of arginine being lower than that required for survival (Rajendran et al., 2016). In view of the importance of TgNPT1 in the survival and virulence of *T. gondii*, we speculate that the mutant strain with a deletion of the *NPT1* gene maybe have the potential to be an attractive candidate for an anti-*T. gondii* vaccine, and whether RH:Δ*NPT1* can be used as an anti-*T. gondii* vaccine needs to be further verified by animal experiments.

In this study, female Kunming mice were immunized with a live attenuated *T. gondii* RH Δ*NPT1* mutant strain to investigate the vaccine’s efficiency by challenging the mice with heterologous strains with different degrees of virulence. The efficacy of immunization with the RH:Δ*NPT1* strain was evaluated by the humoral and cellular immune responses after immunization and the mouse survival rate and cysts burden after acute or chronic infection; in addition, the potential related immune mechanisms of the protection were also addressed.

## Materials and Methods

### Mice

Six-week-old female Kunming mice of specific-pathogen-free (SPF) grade were obtained from the Laboratory Animal Center, Lanzhou Veterinary Research Institute, Chinese Academy of Agricultural Sciences, Lanzhou, Gansu Province, China. As Kunming mice are susceptibility to acute and latent infection with *T. gondii*, they were selected to establish an animal model of infection. According to the regulations of the Administration of Affairs Concerning Experimental Animals, all experiments using animals provided care under standard conditions.

### Parasites

Tachyzoites of *T. gondii* type I (RH), type ToxoDB#9 (PYS), type II (Pru), and RH:Δ*NPT1* mutant strains used in this study were maintained by serial passage in confluent monolayers of human foreskin fibroblasts (HFFs), which were cultured in Dulbecco’s Modified Eagle’s Medium (DMEM) or RPMI-1640 supplemented with 10% fetal bovine serum (FBS). RH:Δ*NPT1* mutant tachyzoites were obtained by deleting the TgNPT1 gene of the *T. gondii* RH strain. Freshly egressed tachyzoites were harvested according to methods described in previous studies and then counted using a hemocytometer (Wang et al., 2016). *T. gondii* Pru cysts were maintained by continuous passage in Kunming mice and isolated from the mouse brain homogenates of infected PRU strains using a previously described strategy (Zhang et al., 2018).

### Preparation of Soluble Tachyzoite Antigens (STAg)

To prepare soluble tachyzoite antigens (STAg), the tachyzoites of the *T. gondii* RH strain were purified as previously described (Li et al., 2014). The suspension of tachyzoites was collected and then washed with PBS. Subsequently, the precipitate was subjected to freeze-thaw cycles followed by sonication and centrifugation at 14,000 *g* for 30 min at 4°C. The supernatant containing the STAg was collected and stored at −80°C.

### Construction of Plasmids and Depletion of *NPT1* in the RH Strain

A CRISPR-Cas9 method was engineered to knockout the *NPT1* gene of the *T. gondii* RH strain. Briefly, to generate a vector of pSAG1:CAS9-U6:sg*NPT1*, single guide (sgRNA) and specific primers (listed in Table 1) were employed to modified the pSAG1:CAS9-U6:sgURPT. Specific primers (listed in Table 1) were designed to amplify the DHFR^*^ resistance cassette. To construct the knockouts, the pSAG1:CAS9-U6:sg*NPT1* and DHFR^*^ resistance cassette were transfected into the tachyzoites of the RH strain as described elsewhere (Wang et al., 2016). Then, the transfected parasites were selected with 3 μM pyrimethamine and the successful integration was confirmed by PCR and RT-PCR analysis using detection primers (KO-*NPT1*-F and KO-*NPT1*-R, see Table 1). The *GRA17* gene was used as positive control to test the quality of cDNA product extracted from different stains, and its primers (*GRA17*-F and *GRA17*-R) were listed in the Table 1. The resultant parasite strain was termed RH:Δ*NPT1*.

**TABLE 1:** Primers used in this study.

**Primer**	**Sequence**
gRNA-P *NPT1*-Fw	5′-GTGCATCGGATACGCTGTTGGTTTTAGAGCTAGAAATAGC-3′
gRNA-R	5′-AACTTGACATCCCCATTTAC-3′
KO-*NPT1*-Fw	5′-GGATATCTGCTTGATACTGTTGGG-3′
KO-*NPT1*-R	5′-TGTTCATCATCAGGAGCTGTTC-3′
DHFR-Fw	5′-GCAGGCTTCAAGCTTCGCCAGGCTGTAAATC-3′
DHFR-R	5′-CTGGGTCGGAATTCATCCTGCAAGTGCATAGAAG-3′
*GRA17*-F	5′-CAATCCAGGGACGAACCATT-3′
*GRA17*-R	5′-TCTGCTTCACGGCCATCTT-3′

### Growth and Virulence of Mutants and WT Strains

Growth assays of the RH:Δ*NPT1* mutant and WT tachyzoites *in vitro* were conducted in 6 well plates containing monolayers of HFF cells within the same or different culture media (DMEM, RPMI-1640 and DMEM + 400 μM arginine medium). The plaque assay was also used to determine whether the *NPT1* gene was deleted in RH strain. The cells were infected with tachyzoites of RH:Δ*NPT1* mutant (∼200 tachyzoites/well) and incubated for 3 h to allow the parasites to enter the host cells. And then washed twice with sterile phosphate-buffered saline to remove unbound parasites. After the fresh medium of DMEM or RPMI-1640 was added to the palate, it was incubated for 7 days at 37°C in 5% CO_2_ environment. The infected HFF cells were fixed with 4% paraformaldehyde in PBS for 15 min, and then incubated with crystal violet staining solution for 10 min at ambient temperature. The size of plaques was determined using inverted microscope as previously described (Wang et al., 2017). To determine the virulence *in vivo* and the immunizing dose, fresh RH or RH:Δ*NPT1* tachyzoites were intraperitoneally injected into mice and then their physical health and mortality status was recorded daily.

### Vaccination of Mice

All of the Kunming mice were randomly divided into two groups (the naive and RH:Δ*NPT1* groups, 46 mice per group). The mice were intraperitoneally injected with parasites at a single dose of 1 × 10^6^ RH:Δ*NPT1* tachyzoites or mock-vaccinated with 200 μl of PBS. For the immunized mice, the potent immune protection efficiency of the RH:Δ*NPT1* was evaluated by challenging the mice with *T. gondii* infection from different stages of the *T. gondii* life cycle.

### Examination of Cytokine Production

Spleens were collected from six mice from each group, either immunized with RH:Δ*NPT1* tachyzoites or PBS, at 42 days post-immunization. Splenocytes from each group were collected as previously described (Li et al., 2014), and their concentrations were adjusted to 3 × 10^6^ cells per milliliter. Then, to measure the cytokine levels, the obtained suspensions were stimulated with 10 μg/ml *T. gondii* soluble tachyzoite antigen (STAg). Culture supernatants were collected and the secreted cytokines levels were measured at different time points by ELISA; the levels of interleukin-2 (IL-2) and IL-4 were evaluated at 24 h post-incubation (hpi), IL-10 at 72 hpi, and IL-12 and IFN-γ at 96 hpi. Each supernatant sample was examined in triplicate using a mouse cytokine kit (eBioscience Bender MedSystems GmbH, Austria) according to the manufacturer’s instructions.

### Measurement of Anti-*Toxoplasma* IgG Antibodies

For the detection of total antibody production and the subclasses of IgG, sera samples were collected from the mouse tail vein after 14, 28, and 42 days post-immunization and stored at −80°C. We coated 96-well microtiter plates with 100 μg (10 μg/ml) of STAg and incubated them at 4°C overnight. Subsequently, every well was washed and blocked as previously described (Xu et al., 2015). After the blood serum samples were diluted 25-fold in PBS, they were added to each well. Then, the plate was incubated at 37°C for 60 min. To detect the bound IgG, IgG1 and IgG2a antibodies, the wells were washed in PBS, and 100 μl of horseradish-peroxidase-conjugated goat anti-mouse IgG (1:250 diluted with PBS), anti-mouse IgG1 (1:500 diluted with PBS), or IgG2a (1:500 diluted with PBS) were added to the wells. Subsequently, a stop solution was added. For quantitation of IgG, IgG1 and IgG2a, their degrees of absorbance were captured by an iMark Microplate Absorbance Reader (Zhu et al., 2017; Wang et al., 2018).

### Protection Against Acute Infection

To determine whether immune protection was activated by immunization with the RH:Δ*NPT1* strain, distinct genotype strains of *T. gondii* were employed to infect the immunized mice. 10 mice of each group was challenged with 1 × 10^3^ tachyzoites of RH or PYS at 42 days post-immunization. The ability of protection against acute infection was determined by the survival rates of all mice (vaccinated and non-vaccinated mice) after infection with strains exhibiting different degrees of virulence. To better understand the effectiveness of immunoprotection, the infected mice were observed and monitored for disease progression for 35 days.

### Protection Against Chronic Infection

Kunming mice were immunized with RH:Δ*NPT1* and then challenged with 20 or 100 cysts of the Pru strain, which was performed as previously described (Zhang et al., 2018). Non-immunized mice were used as controls. And each group contained 10 mice. At 35 days post-challenge, the mice were euthanized. Subsequently, the brains of these animals were collected and ground into homogenate suspensions. The degree of cyst burden in the brain was determined by optical microscopic observation based on counting the number of cysts on 15 slides prepared from each brain homogenate, as previously described (Wang et al., 2018).

### Statistical Analysis

Treatment groups with three or more independent experiments were analyzed for significant differences using Student’s *t*-test or one-way analysis. The significance level was set to 0.05; if values of *P* < 0.05, differences were defined as statistically significant. In the different mouse groups, mouse mortality was expressed by plotting survival curves, and significant differences were determined using the Mantel–Cox log-rank test.

## Results

### Disruption of the *NPT1* Gene in the *T. gondii* RH Strain

**FIGURE 1:**
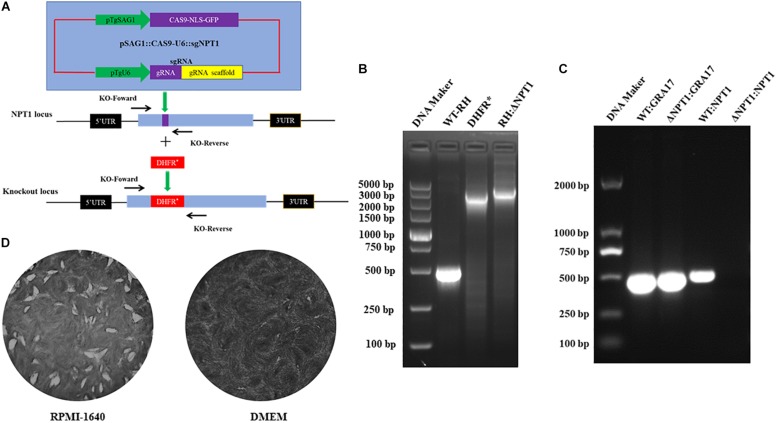
Employment of CRISPR/Cas9 technology to knockout the *NPT1* gene in the *T. gondii* RH strain. **(A)** Schematic illustration of the CRISPR-Cas9 method to disrupt the gene by insertion of the pyrimethamine-resistant DHFR^*^. **(B)** KO-*NPT1*-F and KO-*NPT1*-R were used to amplify the target fragment, which can amplify the coding region in wild-type parasites and a larger fragment in the pyrimethamine-resistant clones. Diagnostic PCR confirming the selection maker DHFR^*^ cassette’s successful integration into the *NPT1* gene (RH:Δ*NPT1*). **(C)** RT-PCR were used to amplify the target fragment, which can amplify the coding region in the wild-type parasites, but did not amplify the targeting aim gene in the *NPT1*-deficiency clones. The *GRA17* gene fragment as the positive control of RT-PCR detected were amplified in wild-type and RH:Δ*NPT1* strain. Diagnostic PCR confirming the selection maker DHFR^*^ cassette successful integration into the *NPT1* gene (RH:Δ*NPT1*). **(D)** Growth analysis of RH:Δ*NPT1-*infected human foreskin fibroblast cells. RH:Δ*NPT1* was normal growth and developed plaque in the RPMI-1640 medium, but its growth capability was remarkably attenuated in the DMEM medium, indicating the successful deletion of the *NPT1* gene.

To delete the *NPT1* gene, the CRISPR-Cas9 plasmids were transduced into tachyzoites of the *T. gondii* RH strain by inserting the selectable maker dihydrofolate reductase (DHFR^*^) into the *NPT1* gene. Then, the knockouts, the pyrimethamine-resistant clones, were successfully generated (Figure 1A). Following selection of the parasite clones, the efficiency of knockout was verified by PCR with specific primers (Figure 1B), and Sanger sequencing of the mutants was performed to affirm that the *NPT1* gene had been knocked out. Meanwhile, the RT-PCR were performed used *GRA17*-F, *GRA17*-R, KO-*NPT1*-Fw and KO-*NPT1*-R (Figure 1C), which also verified that the gene of *NPT1* was successful knockout. As previously reported, the knockouts of the RH strain we obtained had the characteristics of normal growth and proliferation in RPMI 1640 medium and DMEM containing the concentration of arginine present in RPMI-1640 (400 μM), but its growth capability was remarkably attenuated in the DMEM medium (Figure 1D) due to the DMEM medium containing much lower concentrations of arginine compared with RPMI-1640. The WT strain exhibited comparable growth in both environments. The above results revealed that the *NPT1* gene was successfully disrupted in the RH strain.

### Virulence of the Wild-Type and Mutant Strains

**FIGURE 2:**
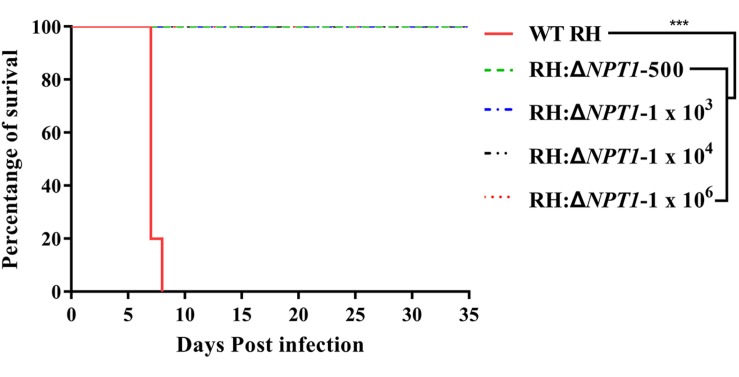
Survival curves of Kunming mice infected with the wild-type *T. gondii* (RH) strain or the RH:Δ*NPT1* strain. The different groups of Kunming mice were challenged with 1 × 10^3^ tachyzoites of the wild-type strain and doses of 500, 1 × 10^3^, 1 × 10^4^, 1 × 10^6^ parasites of the RH:Δ*NPT1* strain. Each group included 10 mice, and the survival time was recorded for 35 days after the challenge (*n* = 10; ^∗∗∗^*P* < 0.001; *n.s.*, not significant; Mantel-Cox-test).

To gain insight into whether RH:Δ*NPT1* was able to be used as an attenuated vaccine, we assessed the virulence of the RH:Δ*NPT1* strain in mice. We observed clearly different survival rates of mice after treatment with the wild-type or mutated strains. Mice were infected with doses of 500, 1 × 10^3^, 1 × 10^4^, or 1 × 10^6^ parasites of the RH:Δ*NPT1* strain, and all of the mice survived the challenge. Furthermore, the mice exhibited no signs of toxoplasmosis, even after infection with high doses of RH:Δ*NPT1* (up to 1 × 10^6^) (Figure 2). Infected-WT RH mice succumbed to infection within 8 days after a dose of 1 × 10^3^ tachyzoites. These results suggested that the virulence of RH:Δ*NPT1* was significantly attenuated compared with the WT, indicating that it might be usable as a live-attenuated vaccine against toxoplasmosis. Based on the health condition and its immune response elicited by different infected does of immunized mice, the vaccinated dose of the RH:Δ*NPT1* strain we selected is 1 × 10^6^.

### Humoral Immune Responses Elicited by RH:Δ*NPT1*

**FIGURE 3:**
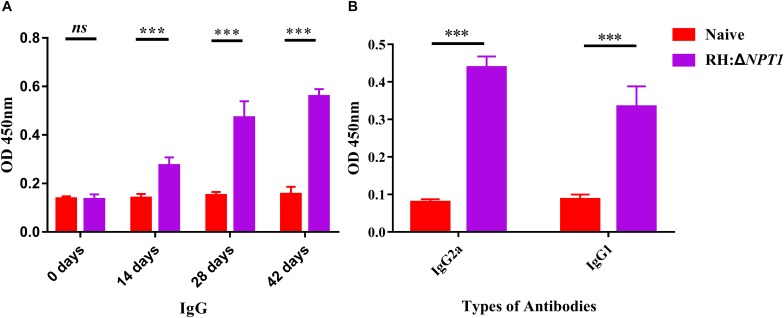
The level of humoral response in the serum of mice immunized with RH:Δ*NPT1*. **(A)** Levels of IgG antibody in the sera obtained from RH:Δ*NPT1*-immunized and non-immunized mice at 0, 14, 28, and 42 days post-vaccination suggest an effective humoral response was elected by the vaccination. **(B)** Levels of IgG subclass (IgG2a and IgG1) antibodies in the sera were collected at 42 days post-immunization, indicating a Th1 response was induced. The average of six biological replicates and three technical replicates are shown. The results are presented as the mean of OD450 ± SD (*n* = 6; ^∗∗∗^*P* < 0.001; *n.s.*, not significant; ANOVA).

To elucidate the potential protection efficiency of this attenuated strain, we measured the level of specific anti-*T. gondii* IgG and IgG isotypes antibodies in the serum of immunized Kunming mice. To establish the experimental model, the mice were vaccinated with 1 × 10^6^ tachyzoites via intraperitoneal injection, and then sera samples were collected from the naive and vaccinated mice. Samples were subjected to ELISA assays to measure their IgG levels at 0, 14, 28, and 42 days post-immunization. In this study, there was a gradual increase in the level of anti-*T. gondii* IgG antibodies after immunization versus naive mice (Figure 3A). Moreover, the titers of IgG reached a peak at 42 days post-immunization. These results suggested that RH:Δ*NPT1* induced a high-level humoral immune response in the immunized mice.

Subsequently, we confirmed that a Th1 or Th2 response was induced by the attenuated vaccine. Serum was collected at 42 days post-immunization to assess the levels of STAg-specific IgG2a and IgG1 isotypes. The serum levels of both IgG2a and IgG1 rose significantly following vaccination, which showed a mixed anti-*T. gondii* Th1/Th2 response elicited by RH:Δ*NPT1* strain infection (Figure 3B). As expected, the augmentation of IgG2a was greater than that of IgG1, which confirmed that a Th1-type response was dominatingly induced.

### Cytokine Measurement After Vaccination

**FIGURE 4:**
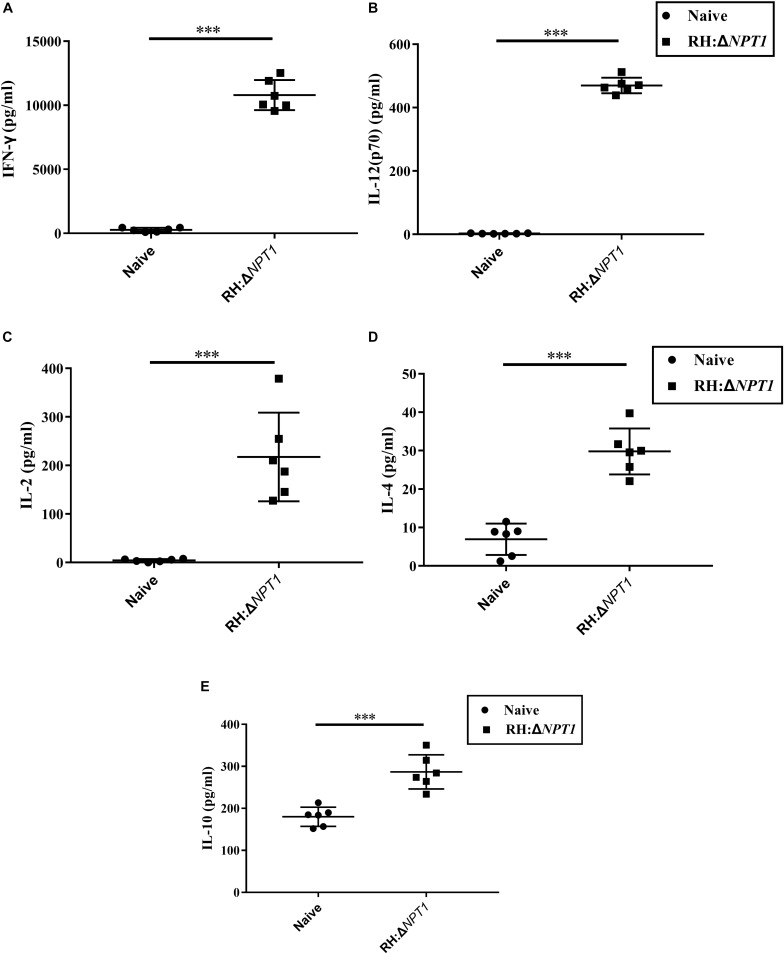
The levels of cytokines in the splenocyte suspension of mice immunized with RH:Δ*NPT1*. Splenocytes collected from the immunized and non-immunized mice (42 days post-vaccination) were co-incubated with STAg (10 μg/ml). Subsequently the levels of Th1 [IFN-γ **(A)**, IL-12 **(B)**, and IL-2 **(C)**] and Th2 [IL-4 **(D)** and IL-10 **(E)**] in the culture supernatants were analyzed by ELISA. The high levels of cytokines produced by splenocytes culture of immunized mice were significantly higher than naive mice. The results are presented as the mean of OD450 ± SD (*n* = 6; ^∗∗∗^*P* < 0.001; *n.s.*, not significant; ANOVA).

To determine whether this clone could induce a sufficient number of cytokines in the immunized mice, the concentration of cytokines, including IFN-γ, IL-2, IL-4, IL-10, and IL-12 in the supernatants of antigen-stimulated splenic cells were determined by ELISA. In the supernatants, significantly higher levels of proinflammatory cytokines (IFN-γ, IL-2, and IL-12), which are essential for protection against intracellular pathogens, were found in the immunized mice relative to the naive mice. IFN-γ was increased the most by immunization (Figures 4A–C). Meanwhile, the production of Th2-associated cytokines (IL-4 and IL-10) by the immunized mice was evidently higher than that in the controls, whereas the augmentation of Th2-associated cytokines were not significantly higher than the Th1-associated cytokines in the vaccinated mice (Figures 4D,E). These findings are consistent with the results that were obtained through the detection of the anti-*T. gondii* IgG isotype, indicating that immunizing mice with RH:Δ*NPT1* could induce a mixed Th1/Th2 response.

### Protection Against Acute Infection

**FIGURE 5:**
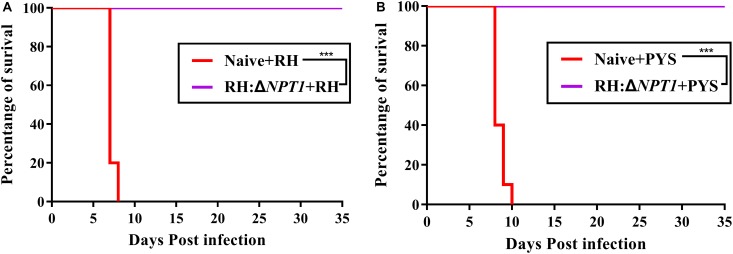
RH:Δ*NPT1* vaccination protected against acute *T. gondii* infection. At 42 days post-immunization, mice were challenged with 1 × 10^3^ tachyzoites of the RH **(A)** and PYS **(B)** strains by intraperitoneal injection. The survival rates of the immunized and non-immunized mice were monitored for 35 days. Survival curves showed the immunized mice had a high survival rate compared with the positive control (non-immunized mice) (*n* = 10; ^∗∗∗^*P* < 0.001; *n.s.*, not significant; Mantel–Cox-test).

Immunoprotection by the attenuated strain was estimated by challenging the mice with 1 × 10^3^ tachyzoites from strains of different degrees of virulence at 6 weeks post-immunization. All mice, including immunized and non-immunized, were observed, including their healthy condition and survival time after challenge. The non-immunized mice infected with PYS and RH parasites exhibited symptoms of toxoplasmosis and died within 10 days post-infection. In contrast, the survival rates of RH:Δ*NPT1*-vaccinated mice challenged with PYS or RH parasites was 100%, and no obvious symptoms were exhibited over the course of 35 days (Figure 5). Therefore, RH:Δ*NPT1* is able to provide efficient protection for the immunized mice against the lethal challenge of PYS or RH parasites.

### Protection Against Chronic Infection

**FIGURE 6:**
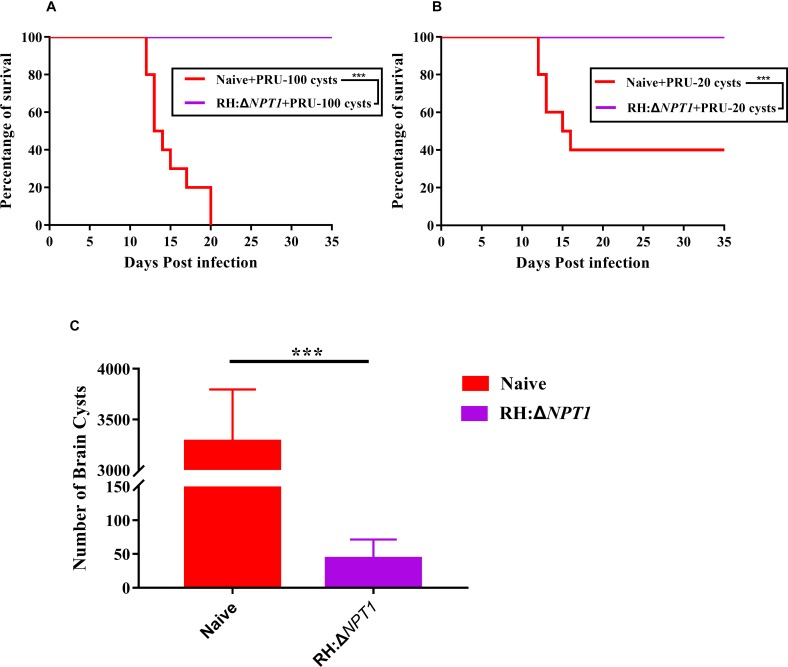
RH:Δ*NPT1* vaccination promotes survival and reduced cysts loads in the brain. **(A,B)** Survival curves derived from mice infected with 20 or 100 cysts of the Pru strain. Compared with non-immunized mice, all of the vaccinated mice survived the challenge (*n* = 10; ^∗∗∗^*P* < 0.001; n.s., not significant; Mantel–Cox-test). **(C)** Protection efficiency of RH:Δ*NPT1* against chronic infection was determined by cyst burden assays. Compared with the non-immunized mice, cyst burden in the brains of immunized mice were significantly reduced after the mice were infected with 20 cysts of the Pru strain. Significance was determined by ANOVA (*n* = 6; ^∗∗∗^*P* < 0.001; *n.s.*, not significant).

To further examine whether the RH:Δ*NPT1* parasites could offer effective protection to mice against chronic infection, we monitored disease progression and cyst burdens in the brains of RH:Δ*NPT1* strain-immunized or naive mice. Throughout the experimental period, the infected-RH:Δ*NPT1* mice that were orally infected with 100 cysts all survived, but the non-infected mice succumbed within 20 days (Figure 6A). When challenged with 20 cysts, the brain cyst burden in the immunized mice (50 ± 28 cysts/brain) was significantly reduced (*P* < 0.05) compared to that in non-immunized mice (3300 ± 453 cysts/brain) (Figure 6C). Some of the controls died after infection with 20 cysts of the *T. gondii* Pru strain (Figure 6B). Interestingly, the immunized mice challenged with 100 cysts not only survived until the experiment was completed but also exhibited fewer cysts in the brain, similar to that in the group infected with 100 cysts (data not shown). Taken together, these results showed the RH:Δ*NPT1* strain is able to provide excellent protection for the immunized mice against *T. gondii* chronic infection.

## Discussion

In light of the fact that *T. gondii* causes serious public health problems and enormous economic losses, safer and more efficient approaches need to be developed to control this ubiquitous pathogen (Dubey, 2010; Zhu et al., 2017). Previous studies have suggested that DNA vaccines, subunit vaccines, inactivated vaccines and secreted antigens could induce a certain level of humoral and cellular responses and prolong the lifespan of vaccinated mice, but none of these vaccines could provide complete protection against further infection (Innes et al., 2011; Ezz Eldin et al., 2015; Yang et al., 2017; Zheng et al., 2017). Although many researchers have performed a tremendous amount of work to pursue effective drugs and better vaccines, a mature product is unlikely to be available in the near future. However, these attempts appear to show that a live-attenuated vaccine may be the best choice. The incomplete strain S48 (Toxovax^®^) has shown promise in reducing the rates of abortion caused by *T. gondii* in sheep. Although this vaccine has some limitations that impede its large-scale application, the success of this attenuated strain displays a more sophisticated requests than other vaccines, and this vaccine should be further validated by experiments to assess its virulence, stability, immunoprotection, and oocyst formation ability (Wastling et al., 1994).

Using the efficient CRISPR-Cas9 strategy to edit genes of *T. gondii* has clearly accelerated the development of attenuated vaccines. This method has been used in the construction of RH:Δ*GRA17*, Pru:Δ*CDPK2* and ME49:Δ*ldh* mutants to explore the possibility of whether the ablation of critical genes in tachyzoites of *T. gondii* could create an ideal vaccine. In this regard, these attempts were successful because the mouse model exhibited an effective protection after vaccination when the mice were faced with a lethal challenge (Shen et al., 2014; Wang et al., 2017, 2018; Xia et al., 2018). Previous studies have shown that *NPT1* is a selective arginine transporter that plays an important role in uptake of cationic amino acids and thus is essential for the survival and virulence of *T. gondii* (Rajendran et al., 2016). When the *NPT1* gene was knocked out in the wild-type *T. gondii*, the mutant strain failed to kill the infected mice, even after infection with 1 × 10^6^ parasites (Rajendran et al., 2016). Based on this observation, we speculated that *NPT1*-defective mutants of a *T. gondii* RH strain might be a live attenuated vaccine candidate to prevent toxoplasmosis. To verify this speculation, we constructed the RH:Δ*NPT1* strain by using the CRISPR-Cas9 method and obtained an effective vaccine candidate against toxoplasmosis.

The RH:Δ*NPT1* strain exhibited some characteristics that meet the prerequisite of an ideal live vaccine. One striking characteristic is that the parasites lacking *NPT1* developed normally within RPMI 1640 medium but could not proliferate in DMEM medium, analogous to what has been described in the drug screening functions of the other edited strains. Acquiring the RH:Δ*NPT1* strain without the need for a drug-screening step makes it easier to produce. If the construction of the RH:Δ*NPT1* strain is adopted for use in inserting other genes, this screening method will avoid the occurrence of drug-resistant strains.

The RH strain of *T. gondii* lack the potential for production of bradyzoite and tissue cyst forming (Asgari et al., 2013), which is an important factor for an ideal attenuated vaccine to leave no cysts in the immunized groups in order to avoid the threat of parasite dissemination via vaccination. For this reason, we decided to start with the RH strain, which probably overcomes some limitations presented by the strains Pru and ME49 (for example, no cyst formation or better immunogenicity), including lack of the risk of transmittance during vaccination or the possibility of a back mutation (which might happen when a cat is vaccinated). Thus, all of these advantages suggest that the RH:Δ*NPT1* strain is more secure than others to use when developing a vaccine.

RH:Δ*NPT1* did not cause any deaths even when the inoculation dose reached 1 × 10^6^ tachyzoites/mouse, which demonstrated that this vaccine has a high biosafety among mice with a normal immunity (Gigley et al., 2009; Fox and Bzik, 2015; Wang et al., 2017, 2018; Xia et al., 2018). However, further research is still required to test whether this attenuated strain can be safely administered to immunocompromised mice.

Specific IgGs are a critical factor in protecting host cells from infection (Sayles et al., 2000; Chen et al., 2017). By detecting levels of specific IgGs in the serum from the vaccinated mice, we demonstrated that RH:Δ*NPT1* could elicit a high level of anti-*T. gondii* IgG antibodies. Moreover, the RH:Δ*NPT1*-vaccinated mice also exhibited a sequential increase in IgG1 and IgG2a, indicating that a Th1/Th2-type mixed immune response was elicited and maintained by immunization. It has been previously reported that IgG2a is more efficient in clearing tachyzoites of *T. gondii* than IgG1 in rodent models (Wang et al., 2018). Thus, in order to better elucidate the humoral immune responses provoked by RH:Δ*NPT1*, we measured the ratio of IgG1/IgG2a by ELISA. We found the level of IgG2a was higher than that of IgG1, suggesting that a prominent Th1 type humoral response was successfully elicited in the vaccinated mice 42 days post-vaccination. If this attenuated strain is considered for use as an ideal commercial vaccine, further research into its ability to provoke long-term immune protection is necessary, similar to the studies conducted on the 1-1 strain (Gigley et al., 2009).

When mice are infected with *T. gondii*, an IFN-γ-dependent cell-mediated immune response is the central contributor to limiting tachyzoite proliferation, while a humoral immune response is induced to a relatively lesser extent (Sasai et al., 2018). This expected trend was observed with this strain. RH:Δ*NPT1*-vaccinated mice produced high levels of Th1-associated cytokines (IFN-γ, IL-2 and IL-12), which can trigger multiple intracellular mechanisms to control and kill tachyzoites by activating cytotoxic lymphocytes and macrophages, leading to effective protection against *T. gondii* acute infection (Khan et al., 1994; Gazzinelli et al., 1996; Sa et al., 2013). We also found that the levels of Th2-biased cytokines (IL-4 and IL-10) were increased. IL-10 and IL-4 are important negative regulators of inflammatory responses during the course of infection with *T. gondii*, which are sufficient to provoke a Th1 immune response to avoid host mortality associated with the development of severe immunopathology (Gazzinelli et al., 1996; Roberts et al., 1996; Suzuki et al., 2000). IL-4 can modify intracellular replication and prevent cyst formation in the brain, and thus, the high level of IL-4 may shed light on the reduced number of brain cysts in chronic infection (Suzuki et al., 1996).

Considering the results of the elevated levels of IgG and cytokines in the immunized mice, we concluded that RH:Δ*NPT1* could confer effective protection for mice against a lethal challenge. As anticipated, the non-immunized mice were euthanized due to severe illness within 7–10 days post-infection, whereas all immunized mice survived, indicating that vaccination with RH:Δ*NPT1* is able to achieve complete protection of mice against challenge with strains of different degrees of virulence. It is worth noting that compared with most existing vaccines, RH:Δ*NPT1* can prevent damage in many aspects, such as the death caused by high-dose infection, the incomplete protection caused by low-dose infection, and the formation of cysts (Wang et al., 2018).

To test the protective efficacy of the RH:Δ*NPT1* mutant strain against chronic infection, the vaccinated mice were orally infected with 20 or 100 cysts of type II Pru. Consistent with the results of the cytokines, this vaccination severely reduced the parasite cyst burden and potentially prevented cyst formation in the brain of the vaccinated mice as a means of enhancing protection after ingestion of a high dose of type II cysts. However, there was the somewhat unexpected fact that vaccination could not completely prevent cyst formation in the chronic infection experiment, and a few brain cysts were observed in the mice vaccinated with the RH:Δ*NPT1* strain. To improve its protective effect against cyst formation after infection with a type II strain, the vaccine needs to be further developed to focus on other strains, such as Pru and ME49, as material to confirm the efficiency of various attenuated vaccines (Wang et al., 2018; Xia et al., 2018). For preparation of a live-attenuation vaccine, we suggest using different endemic strains with distinct capabilities of pathogenicity to confirm the direct role of vaccination in immunity.

## Conclusion

In conclusion, in this study, we constructed a RH:Δ*NPT1* mutant strain and evaluated its potential as a live attenuated vaccine against *T. gondii* infection. The results demonstrated that vaccination with the RH:Δ*NPT1* mutant strain can elicit high levels of humoral and cellular immune responses and provide effective immune protection against infection in mice. RH:Δ*NPT1* not only can completely protect against acute infection from various genotype strains of *T. gondii* but also can improve the survival rate and reduce the brain cyst burden in response to chronic infection, suggesting that the RH:Δ*NPT1* strain is a potential and promising live-attenuated vaccine against toxoplasmosis. Further work should evaluate its protective efficacy in food-producing animals and in the definitive feline host.

## Data Availability

All datasets generated for this study are included in the manuscript.

## Ethics Statement

This study was approved by the Animal Administration and Ethics Committee of Lanzhou Veterinary Research Institute, Chinese Academy of Agricultural Sciences. All mice were cared for and handled in accordance with the regulations of the Good Animal Practice requirements of the Animal Ethics Procedures and Guidelines of the People’s Republic of China for animal experimentation. All efforts were made to minimize animal suffering and to reduce the numbers of animals used in the experiments.

## Author Contributions

D-HZ, J-LW, and X-QZ conceived the project, designed the experiments, and critically revised the manuscript. W-BY, J-LW, QG, and KC performed the experiments and analyzed the data. W-BY and J-LW drafted the manuscript. YZ, QL, and Q-LL helped in the implementation of the experiments. All authors reviewed and approved the final version of the manuscript.

## Conflict of Interest Statement

The authors declare that the research was conducted in the absence of any commercial or financial relationships that could be construed as a potential conflict of interest.
